# Predictive factors of 5-year relapse-free survival in HR+/HER2- breast cancer patients treated with neoadjuvant endocrine therapy: pooled analysis of two phase 2 trials

**DOI:** 10.1038/s41416-020-0733-x

**Published:** 2020-01-31

**Authors:** Florence Lerebours, Marina Pulido, Emmanuelle Fourme, Marc Debled, Véronique Becette, Hervé Bonnefoi, Sofia Rivera, Gaetan MacGrogan, Marie-Ange Mouret-Reynier, Christine Tunon de Lara, Jean-Yves Pierga, Christel Breton-Callu, Laurence Venat-Bouvet, Simone Mathoulin-Pélissier, Thibault de la Motte rouge, Florence Dalenc, Brigitte Sigal, Thomas Bachelot, Jérôme Lemonnier, Nathalie Quenel-Tueux

**Affiliations:** 1 Institut Curie, Saint Cloud, France; 2 Clinical and Epidemiological Research Unit, Institut Bergonié, INSERM CIC 14.01, Bordeaux, France; 3 Institut Bergonié, Bordeaux, France; 4 Institut Curie, Paris, France; 5 Department of Radiation Oncology, Gustave Roussy Cancer Campus, Villejuif, France; 6 Centre Jean Perrin, Clermont-Ferrand, France; 7 CHU, Limoges, France; 8 Centre Eugène Marquis, Rennes, France; 9 Institut Claudius Regaud-IUCT Oncopole, Toulouse, France; 10 Centre Léon Bérard, Lyon, France; 11 R&D UNICANCER, UCBG, Paris, France

**Keywords:** Breast cancer, Risk factors, Breast cancer

## Abstract

**Background:**

Few data are available on survival and predictive factors in early breast cancer (BC) patients treated with neoadjuvant endocrine therapy (NET).

**Methods:**

This is a pooled analysis of two multicentre, randomised non-comparative phase 2 clinical trials evaluating neoadjuvant anastrozole and fulvestrant efficacy for postmenopausal HR+/HER2- breast cancer patients: HORGEN (NCT00871858) and CARMINA02 (NCT00629616) studies.

**Results:**

In total, 236 patients were included in CARMINA02 and HORGEN trials. Modified intention-to-treat analysis was available for 217 patients. Median follow-up was 65.2 months. Relapse-free survival (RFS) and overall survival (OS) at 5 years were 83.7% (95% CI: 77.9–88) and 92.7% (95% CI: 88.2–95.6), respectively, with no difference between treatment arms. On univariate analysis, tumour staging (T2 vs T3–4; *p* = 0.0001), Ki-67 at surgery (≤10% vs >10%; *p* = 0.0093), pathological tumour size (pT1–2 vs pT3–4; *p* = 0.0012) and node status (pN negative vs positive; *p* = 0.007), adjuvant chemotherapy (*p* = 0.0167) and PEPI score (PEPI group I + II vs III; *p* = 0.0004) were associated with RFS. No events were observed in patients with pathological response according to the Sataloff classification. Multivariate analysis showed that preoperative endocrine prognostic index (PEPI) group III was associated with significantly worse RFS (*p* = 0.0069, hazard ratio = 3.33 (95% CI: 1.39–7.98)).

**Conclusions:**

Postmenopausal HR+/HER2- breast cancer patients receiving NET generally have a favourable outcome. The PEPI score identifies a subset of patients of poorer prognosis who are candidates for further additional treatment.

## Background

Pathological complete response (pCR) after neoadjuvant chemotherapy (NCT) is correlated with prognosis, although differences are observed among breast cancer subtypes.^1^ International guidelines consider that neoadjuvant endocrine therapy (NET) given for 4–8 months is a validated treatment in postmenopausal women presenting a hormone receptor-positive (HR+)/HER2-negative (HER2-) breast cancer to improve surgical outcome and allow breast-conserving surgery (BCS) (https://www.nccn.org/professionals/physician_gls/).^2,3^ In this population, NET may be more efficient than chemotherapy, with a lower toxicity profile.^4^

Few data are available on survival of patients treated with NET. Understanding the link between tumour response to NET and relapse risk would help clinicians to make decisions about additional treatment options for patients treated with NET. Although pCR is uncommon after NET, prognosis seems favourable in most cases.^5,6^ Ki-67 expression, before and especially under endocrine treatment, has been shown to predict relapse-free survival (RFS) in the IMPACT trial.^7,8^ Based on patients included in the P024 trial, Ellis et al. developed a preoperative prognostic index (PEPI score) validated in an independent cohort of patients from the IMPACT study.^5,9,10^ It combines the post-treatment Ki-67 level with ER status, pathological tumour size and nodal status.

We published in 2015 and 2016 results of two sister phase 2 studies evaluating anastrozole and fulvestrant as NET in postmenopausal HR+/HER2- breast cancer patients (CARMINA02 NCT00629616; HORGEN NCT00871858).^6,11^ Population and design of both studies were similar. Clinical response rate at 6 months was the primary endpoint in both studies: −58.9% in the anastrozole arm and 53.8% in the fulvestrant arm in HORGEN; 52.6% and 36.8% in CARMINA02. Most secondary endpoints were the same, in particular pathological response rates, PEPI score evaluation and survival data. These two trials have now sufficient follow-up to investigate, in a pooled analysis, the relationships between the baseline and post-NET tumour characteristics, and 5-year RFS.

## Methods

### Study design and procedures

This is an exploratory prognostic, pooled analysis of two multicentre, randomised (1:1) non-comparative phase 2 clinical trials evaluating neoadjuvant anastrozole and fulvestrant efficacy for postmenopausal HR+/HER2- breast cancer patients: HORGEN and CARMINA02 (UCBG 0609) studies that have been previously published.^6,11^ In both trials, clinical response rate according to RECIST v1.1 was the primary endpoint.^12^ RFS and OS at 5 years were secondary endpoints in both trials.

Both trials were conducted in accordance with the Declaration of Helsinki and Good Clinical Practice guidelines. All patients gave written informed consent. These studies were authorised by the French Health Authority, and were approved by the local ethics committee.

Eligibility criteria for both studies were postmenopausal patients with histologically confirmed, untreated, invasive, HR+/HER2- (HER2+ allowed in HORGEN), operable, T2–T4 (non-inflammatory), N0–N3 (CARMINA02) or N0–N1 (HORGEN), non-metastatic (M0) breast cancer. Patients with histological grade III tumours were non-eligible if <65 years old. In the present pooled study, patients from HORGEN study with HER2+ disease at inclusion were excluded, as well as patients from CARMINA02 with N2–N3 node status.

In both studies, eligible patients were randomised to receive anastrozole (per os, 1 mg daily) or fulvestrant (intramuscular, 500 mg on D1, D15 and D29 and then every 4 weeks) for 4–6 months before surgery. Each centre decided on adjuvant treatment according to the local policy.

For the pooled analysis, the following procedures have been employed: clinical response was assessed by using RECIST v1.1.^12^ Pathological response was assessed using the Sataloff classification,^13^ with objective response defined as TA or TB combined with NA or NB. Ki-67 was scored centrally in a blinded manner, according to the recommendations of Dowsett et al., by counting at least 1000 tumour nuclei per sample after staining with mib1 antibody.^14^ The PEPI score was calculated by combining pT, pN, ER Allred score and Ki-67 at surgery, as previously published.^5^

The primary endpoint of this pooled analysis was 5-year RFS in each treatment arm. Secondary endpoints included 5-year OS, prognostic factors of 5-year RFS and overall safety of the two pooled studies.

The following prognostic factors of RFS were explored: age (≤70 years vs >70), tumour (T2 vs T3–T4) and node (N0 vs N1) staging, histological type (ductal vs other), histological grade (I vs II+III), treatment arm (anastrozole vs fulvestrant), clinical response (CR+PR vs SD+PD), pathological response according to the Sataloff classification (pathological response defined as TA or TB+NA or NB), baseline and surgical ER Allred score (6–8 vs other) and Ki-67 expression (≤10% vs >10%), pathological tumour size (pT1–2 vs pT3–4), node status (pN negative vs positive), preoperative endocrine prognostic index (PEPI) group (groups I (score 0) +II (score < 4) vs group III (score ≥ 4)), adjuvant chemotherapy (no vs yes) and study (CARMINA02 vs HORGEN).

### Statistics

RFS was measured on the modified intention-to-treat (mITT) population from the date of randomisation to the date of the following events, whichever occurred first:^15^ invasive ipsilateral breast tumour recurrence/progression; local invasive recurrence/progression; regional invasive recurrence/progression; appearance/occurrence of metastatic recurrence; death (of any cause). mITT is defined as all randomised eligible patients who have started their allocated treatment. Patients were analysed in the arm they were allocated to. Exclusions from primary analysis had been assessed by the Steering Committee of the pooled study. Secondary endpoints were assessed on mITT population. OS was defined as the delay between the start date of treatment and the date of death (of any cause). Safety was assessed on all participants who have started their allocated treatment using the CTCAE v3.0 from the National Cancer Institute.

Quantitative variables were described by using mean and standard deviations (normality assumption satisfied). Other descriptive statistics (median, range and quartiles) were also used. Qualitative variables were described using frequency, percentage and 95% confidence interval (95% CI, binomial law).

Survival endpoints were analysed using the Kaplan–Meier (K–M) method. The median survival rates were reported with their 95% CI. Median follow-up was calculated using the reverse K–M method. Multivariate analyses were conducted based on Cox’s proportional risk method, and after checking the risk proportionality hypothesis. Univariate Cox regression analysis was performed first to assess the association between each variable and RFS, followed by multivariate Cox regression analysis after selecting factors from univariate regressions that showed statistically significant association with survival with *p* < 0.15. For the final multivariate model, only factors statistically significant at *p* < 0.05, based on the Wald or likelihood ratio test if appropriate, after adjustment for the other factors, will then be introduced in the final model. Stepwise multivariable regression analysis with backward selection was also performed.

## Results

### Trial conduct and patient’s cohort

**Fig. 1:**
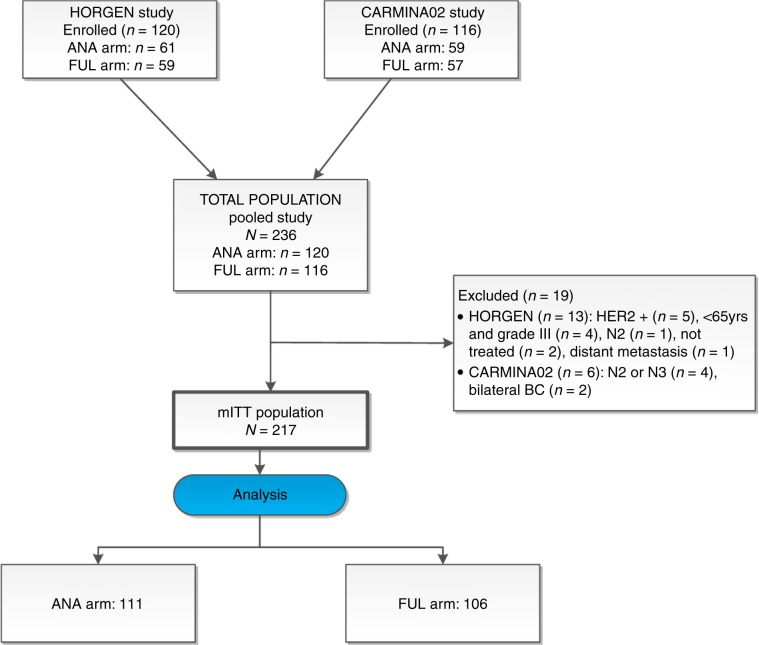
Study flowchart.

From October 2007 to April 2013, 236 patients were included, 116 in CARMINA02 and 120 in HORGEN. mITT analysis was available for 217 patients. In total, 19 patients were excluded: 6 from CARMINA02 (4 staged N2 or N3 and 2 bilateral breast cancer) and 13 from HORGEN (5 HER2-positive tumours, 4 patients <65 years old with grade III tumours, 1 staged N2, 1 with distant metastases at diagnosis and 2 patients not treated) (study flowchart, Fig. 1). In total, 111 patients received anastrozole (ANA) and 106 fulvestrant (FUL). Baseline characteristics of the 217 patients were well balanced between treatment arms (Table 1).

**Table 1:** Baseline characteristics of patients.

	Anastrozole *n* (%)	Fulvestrant *n* (%)
Age		
≤70 years	59 (53.2)	46 (43.4)
>70 years	52 (46.8)	60 (56.6)
*T*		
T2	87 (78.4)	73 (68.9)
T3	13 (11.7)	18 (17.0)
T4	11 (9.9)	15 (14.1)
*N*		
N0	77 (69.4)	67 (63.2)
N1	34 (30.6)	39 (36.8)
Histological type		
Ductal	73 (65.8)	63 (59.4)
Lobular	34 (30.6)	38 (35.9)
Other	4 (3.6)	5 (4.7)
Histological grade		
Grade 1	21 (18.9)	21 (19.8)
Grade 2	82 (73.9)	73 (68.9)
Grade 3	5 (4.5)	10 (9.4)
NA	3 (2.7)	2 (1.9)
Baseline ER Allred score		
4–5	1 (0.9)	2 (1.9)
6–8	110 (99.1)	104 (98.1)
Baseline Ki-67		
≤10%	38 (34.2)	34 (32.1)
>10%	56 (50.5)	52 (49.0)
NA	17 (15.3)	20 (18.9)

*NA* not available.

### Clinical response and surgery outcome

An objective clinical response was observed in 62 (55.9%) patients in the ANA arm and 47 (44.3%) in the FUL arm. Based on clinical response, 211 patients underwent surgery after 4–6 months of NET. BCS was done in 67 (60.4%) patients in the ANA arm and 56 patients (52.8%) in the FUL arm (Table 2). No pCR was observed. A pathological response according to the Sataloff classification was observed in 20 (18%) and 14 (13.2%) patients in the ANA and FUL arms, respectively (Table 2). The distribution of the whole population according to the Sataloff classification is provided as Supplementary Table 1.

**Table 2:** Type of surgery and tumour characteristics.

	Anastrozole *n* (%)	Fulvestrant *n* (%)
Surgery		
Breast-conserving surgery	67 (60.4)	56 (52.8)
Radical mastectomy	44 (39.6)	44 (41.5)
None	–	6 (5.7)
pT		
pT1/T2	100 (90.1)	77 (72.6)
pT3/T4	9 (8.1)	16 (15.1)
NA	2 (1.8)	13 (12.3)
pN		
Negative	54 (48.6)	42 (39.6)
Positive	55 (49.6)	53 (50)
NA	2 (1.8)	11 (10.4)
Pathological response^a^		
Non-responder	86 (77.5)	80 (75.5)
Responder	20 (18.0)	14 (13.2)
NA	5 (4.5)	12 (11.3)
Allred ER score		
0–5	1 (0.9)	18 (16.9)
6–8	102 (91.9)	72 (67.9)
NA	8 (7.2)	16 (15.2)
Ki-67		
≤10%	72 (64.9)	65 (61.3)
>10%	22 (19.8)	21 (19.8)
NA	17 (15.3)	20 (18.9)
PEPI group		
I and II (<4)	56 (50.5)	48 (45.3)
III (≥4)	36 (32.4)	30 (28.3)
NA	19 (17.1)	28 (26.4)

TA combined with NA or NB and TB with NA or NB indicate an objective pathological response.

*NA* not available, *PEPI* preoperative endocrine prognostic index.

^a^according to the Sataloff classification.^13^

*NA* not available, *PEPI* preoperative endocrine prognostic index.

In total, 21.7% of patients received adjuvant chemotherapy (25 in ANA and 22 in FUL arm).

Thirty seven Ki-67 expression levels at surgery were missing, precluding the evaluation of the PEPI score in those cases. The PEPI score was available for 170 patients (92 in ANA arm and 78 in FUL arm). Seventeen patients (18.5%) in ANA arm and 10 (12.8%) in FUL arm had a PEPI score 0 (group I), 39 (42.4%) and 38 (48.7%) a score <4 (group II) and 36 (39.1%) and 30 (38.5%) a score ≥4 (group III) (Table 2). The breakdown for each score for anastrozole and fulvestrant is provided as Supplementary Table 2.

Information on both the PEPI score and adjuvant chemotherapy was available for 159 patients. No adjuvant chemotherapy was performed in 22 patients in PEPI group I, whereas 9 of 75 (12%) and 30 of 62 (48.4%) patients in PEPI groups II and III, respectively, received adjuvant chemotherapy.

### Safety

The safety data were available for 234 patients, 119 in arm A and 115 in arm B (Table 3).

**Table 3:** Number of patients that experienced adverse events at least once during studies.

	Grade 1, 2		Grade 3	
Adverse event	Anastrozole *n* (%)	Fulvestrant *n* (%)	Anastrozole *n* (%)	Fulvestrant *n* (%)
Asthenia	16 (6.8)	29 (12.4)	1 (0.4)	0 (0)
Headache	6 (2.6)	5 (2.1)	0 (0)	0 (0)
Hot flashes	29 (12.4)	19 (8.1)	0 (0)	3 (1.3)
Injection site reaction	0 (0)	14 (6.0)	0 (0)	0 (0)
Nausea	5 (2.1)	5 (2.1)	0 (0)	0 (0)
Pain (muscular/bone/joint)	36 (15.4)	27 (11.5)	2 (0.9)	1 (0.4)
Weight gain	5 (2.1)	2 (0.9)	0 (0)	0 (0)

The percentages presented were calculated based on the total number of treated patients (*n* = 234). The adverse events were presented according to the CTCAE v3.0.

Toxicities were grade 1 or 2 muscular/bone/joint pain, hot flashes, asthenia, nausea, headache, weight gain and injection site reaction for fulvestrant. Grade 3 hot flashes, pain or asthenia were observed in seven patients. No grade 4 toxicity was observed.

### Survival analysis

Median follow-up was 65.2 months (95% CI: 64.4–66).

In total, 39 events (18.0% of the mITT population) were observed: 18 deaths (8.3% of mITT population) (8 in ANA arm and 10 in FUL arm) including 8 deaths from breast cancer relapse, 3 from other primary cancer, 1 post-surgical heart failure and 6 from unknown cause, and 30 relapses (13.8% of mITT population) (15 in ANA arm and 15 FUL arm) including 20 distant metastases, 4 loco-regional relapses, 3 both loco-regional and metastatic relapses and 3 disease progressions.

RFS and OS at 5 years were 83.7% (95% CI: 77.9–88.0) and 92.7% (95% CI: 88.2–95.6), respectively. No difference was observed between treatment arms.

### Prognostic factors

Univariate and multivariate analyses are shown in Table 4. On univariate analyses, tumour staging (T3–4 vs T2; *p* = 0.0001), Ki-67 at surgery (>10% vs ≤10%; *p* = 0.0093), pathological tumour size (pT3–4 vs pT1–2; *p* = 0.0012), node status (pN positive vs negative; *p* = 0.0004), adjuvant chemotherapy (yes vs no; *p* = 0.0167) and PEPI group (III vs I + II; *p* = 0.0004) were associated with RFS.

**Table 4:** Prognostic factors of RFS; univariate and multivariate analyses.

	Univariate analysis		Multivariate analysis	
Covariate	*p* value	Hazard ratio [95% CI]	*p* value	Hazard ratio [95% CI]
Age (ref ≤70 years old)	0.6025	0.85 [0.45; 1.59]		
Tumour staging (ref T2)	**0.0001**	**3.46 [1.84; 6.49]**	NS	
Node staging (ref N0)	0.3808	1.33 [0.70; 2.55]		
Histological type (ref ductal)	0.3620			
Lobular carcinoma		1.54 [0.81; 2.93]		
Other		0.71 [0.10; 5.30]		
Histological grade (ref I)	0.9640	0.98 [0.45; 2.14]		
Baseline ER Allred score (refs. ^5–7^)	0.9899	^a^		
Baseline Ki-67 (ref ≤10%)	**0.0681**	**2.12 [0.95; 4.73]**	NS	
Treatment (ref anastrozole)	0.4090	1.30 [0.69; 2.45]		
Study (ref CARMINA02)	**0.1326**	**1.63 [0.86; 3.10]**	NS	
Clinical response (ref OR)	0.3136	1.38 [0.73; 2.61]		
Pathological response (ref TA or TB and NA or NB)	0.9846	^b^		
Pathological tumour size (ref pT1–2)^c^	**0.0012**	**3.36 [1.61; 7.00]**		
Node status (ref pN negative)c	**0.0007**	**4.22 [1.84; 7.00]**		
ER Allred score at surgery (refs. ^5–7^)^c^	**0.1340**	**2.07 [0.80; 5.39]**		
Ki-67 at surgery (ref ≤10%)^c^	**0.0093**	**2.61 [1.27; 5.38]**		
Adjuvant chemotherapy (ref no)	**0.0167**	**2.27 [1.16–4.43]**	NS	
PEPI group (refs I and II)	**0.0004**	**4.40 [1.94; 10.00]**	**0.0069**	**3.33 [1.39; 7.98]**

Bold values indicate variables statistically significant with *p* value  <  0.15 (backward selection) included in the multivariate analysis. Since the proportionality assumption was not satisfied for the variable ‘Pathological response’, multivariate analysis was stratified on this variable.

*ref* reference, *OR* objective response as per RECIST 1.1, *NS* not statistically significant, *PEPI* preoperative endocrine prognostic index.

^a^Hazard ratio not calculable because of unbalanced figures (only two ER Allred scores <6–8).

^b^Hazard ratio not calculable (no events were observed in pathological responders).

^c^Factors accounted for in the ‘PEPI group’ variable.

This last finding indicates that patients assigned to PEPI group III have a significantly higher risk of death or relapse than patients assigned to PEPI groups I–II. Since PEPI appears to have a prognostic effect on RFS, it has been decided to not include its components (pN, pT, Ki-67 and ER Allred score at surgery), although three of them are significant. Indeed, PEPI group is a variable that alone summarises the information of these four variables, and since 39 events were observed, the number of potential prognostic factors to include in the multivariable model must be minimised, due to a lack of power. It should be noted that, since only three tumours had baseline ER Allred score <6, this variable was not investigated in univariate analysis.

The proportionality assumption was not satisfied for the variable ‘Pathological response’, since no event was observed in patients with pathological response (*n* = 34, 15.7%). Hence, multivariate regression model was stratified on this variable.

**Fig. 2:**
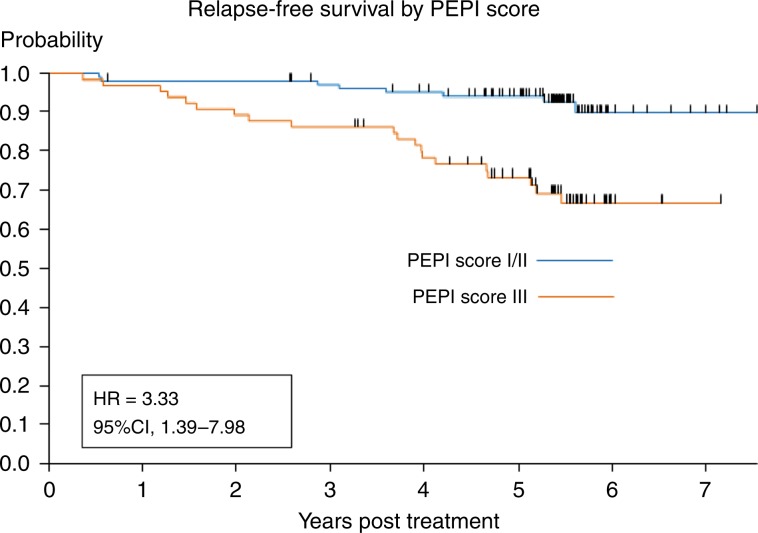
RFS curve according to the PEPI groups. PEPI group I: PEPI score = 0; PEPI group II: score <4; PEPI group III: score ≥4.

The final multivariate model, including covariates with *p* value < 0.15 other than those that compose the PEPI score (tumour staging, baseline Ki-67, study, adjuvant chemotherapy and PEPI score), showed that PEPI group III was associated with significantly worse RFS (*p* = 0.007, hazard ratio = 3.33 [1.39; 7.98]). RFS curves according to the PEPI groups I+II vs III are shown in Fig. 2. The Kaplan–Meier plot for all three groups is provided as Supplementary Data (Supplementary Fig. 1).

Of note, a similar number of events were observed for patients in PEPI group III whether or not they received adjuvant chemotherapy (10 and 9 events, respectively).

## Discussion

In a selected population of postmenopausal patients with HR+/HER2- breast cancer, NET is efficient to improve surgical outcome and better tolerated compared with chemotherapy.^4^ Phase 3 NET trials have demonstrated clinical response rates in approximately half of patients with anti-aromatases, and an improvement of up to 40% of BCS rates.^9,10,16,17^ Nevertheless, few data are available on both survival and prognostic factors in these patients. Very little is known about the population that will require additional treatments.

pCR is considered a positive prognostic factor in breast cancer patients treated with NCT. Of note, the magnitude of the benefit from this pCR varies according to molecular subtype of the breast tumour.^1^

The pCR rate ranges 1–15% in patients treated with NET. At surgery, after 4–6 months of NET, tumours often remain large with persistent node involvement when initially present.^4,18,19^ Despite this, the prognosis is often good.^5,6,19^ We report similar results in this study. With a median follow-up of more than 5 years, 30 relapses, of which 4 loco-regional only, and 8 deaths from breast cancer were observed. Longer follow-up may be required as HR+ breast cancer patients tend to experience late relapses.^20^ Both CARMINA02 and HORGEN trials were non-comparative trials thus not designed to assess the superiority of either of the endocrine treatments in terms of clinical response. First-line treatment with fulvestrant gave longer progression-free survival compared with anastrozole in patients with advanced breast cancer in the FALCON trial.^21^ We did not observe any survival difference between anastrozole and fulvestrant given as neoadjuvant treatments.

The clinical response has been often used as the primary endpoint in NET trials. However, this assessment may be imprecise.^6,8^ Surgical outcome as an endpoint requires a baseline evaluation of the rate of radical surgery, and eligibility for BCS is subjective.^6,17^

Assessment of pathological response is more precise than that of clinical response, although measurement of pathological tumour size may also be biased. However, pathological examination may also estimate the percentage of residual cancer cellularity.^13,22,23^ Both CARMINA02 and HORGEN trials chose the Sataloff classification to assess the pathological response frequency and prognostic value after NET. It should be underlined that the 34 patients who achieved a pathological response were event-free. Hence, we chose to stratify Cox model on this variable. Nevertheless, one limitation of stratified models is that there is no way to carry out inference for the stratification variable, so it was impossible to estimate the prognostic value of the pathological response.

An alternate endpoint in NET trials is the Ki-67 expression score at baseline and under treatment, either after 2–4 weeks or at surgery.^24^ It has been shown to correlate with long-term outcome especially under treatment.^5,7,24^

Survival as an endpoint for NET may be confounding because of the use of adjuvant chemotherapy and/or endocrine therapy, and because of late relapses in HR+ breast cancer patients.^4,20^

The PEPI score, developed within the P024 trial and validated in an independent population from the IMPACT trial, combines assessment of pathological staging, the residual ER Allred and Ki-67 scores.^5,9,10^ Since fulvestrant downregulates ER expression, a modified PEPI score (mPEPI score) including T, nodes and Ki-67 without ER, is being evaluated in the ALTERNATE (NCT01953588) trial that compares neoadjuvant anastrozole vs fulvestrant vs anastrozole +fulvestrant. Because of its ongoing prospective assessment, we did not use the modified PEPI score for analyses. Moreover, only one tumour had an ER Allred score 0–2 at surgery, which would have no impact on the prognostic analyses.

In this pooled analysis, PEPI group III (score ≥4) compared with PEPI groups I or II was the only variable significantly associated with worse 5-year RFS on multivariate analysis. Unlike the results of ACOSOG Z1031 trial, no statistically significant survival differences were found between patients with a PEPI score 0 (group I) and those with PEPI score > 0, although a trend was observed (*p* = 0.06, data not shown).^24^ This may be due to the sample size since only 27 patients had a PEPI score 0. Patients of PEPI group III should be offered post-neoadjuvant treatments such as chemotherapy. Unfortunately, chemotherapy has poor efficacy in patients eligible for NET, i.e. with ER-rich and low-proliferating tumours.^24^ In the present analysis, ten events were observed for patients in PEPI group III who underwent adjuvant chemotherapy and nine for patients in PEPI group III who did not receive adjuvant chemotherapy, suggesting that the addition of PEPI score analysis may not change the patient’s outcome. Given the efficacy of CDK4/6 inhibitors in a metastatic setting, NET could be combined with these targeted therapies like those in the NeoPALAna, PALLET or NeoPal trials.^25–29^ The NeoPal trial (NCT02400567) compared anthracycline- and taxane-based chemotherapy with the association of letrozole and palbociclib as neoadjuvant treatments in high-risk luminal breast cancers.^29^ Both treatment arms led to poor pathological response rates, but clinical and biomarker responses were encouraging with letrozole–palbociclib combination, with a much more favourable safety profile. Survival data and PEPI score of NeoPal are under investigation.

The PEPI score was previously shown to be the only factor correlated with 3-year RFS in the CARMINA02 trial.^6^ Its prognostic role was also validated in patients from the IMPACT and ACOSOG Z1031 trials.^5,24^

### Conclusions

Postmenopausal HR+/HER2- breast cancer patients receiving NET generally have a favourable outcome. This study further validates the PEPI score as a tool to identify a subset of patients with poorer prognosis who are candidates for further additional treatments. We encourage the use of this score in NET clinical trials as well as ‘real-life’ setting.

## Supplementary information

Supplementaryi files

Supplementaryi legends

## Data Availability

Data supporting the results reported in the article can be found at UNICANCER datacenter in Montpellier, France for CARMINA02 study, and in Bergonié Institute, Bordeaux, France for HORGEN study. Data analysis has been performed in the Clinical and Epidemiological Research Unit, Institut Bergonié, INSERM CIC 14.01, Bordeaux, France.
