# Supplementary material for: Predictive factors of 5-year relapse-free survival in HR+/HER2- breast cancer patients treated with neoadjuvant endocrine therapy: pooled analysis of two phase 2 trials
Source: Br J Cancer. 2020 Jan 31;122(6):759–65. doi: 10.1038/s41416-020-0733-x (PMC7078275; doi:10.1038/s41416-020-0733-x)
Supplement: Supplementary file 1 — Supplementaryi files [file 41416_2020_733_MOESM1_ESM.docx]

**Supplemental Figure 1**


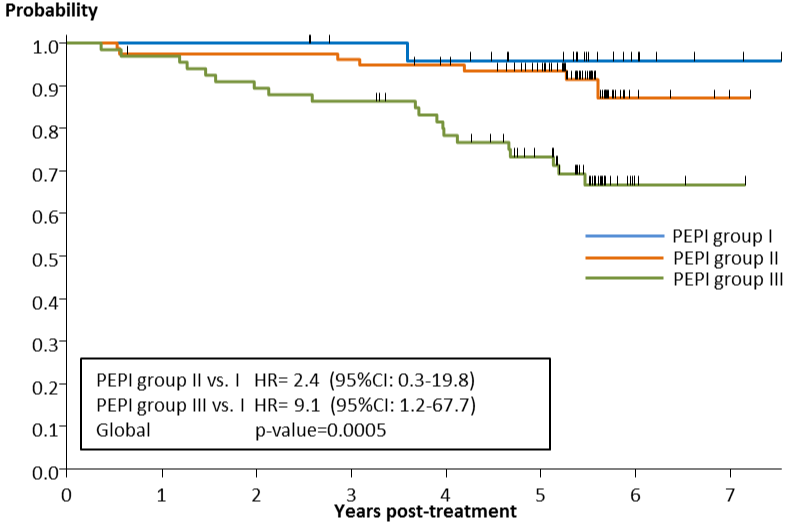


**Supplemental table 1**

|  | Anastrozole | | Fulvestrant | |
| --- | --- | --- | --- | --- |
|  | N | % | N | % |
| **Sataloff’s classification T** |  |  |  |  |
| . | 5 | 4.5 | 12 | 11.3 |
| T A | 1 | 0.9 | 1 | 0.9 |
| T B | 40 | 36.0 | 22 | 20.8 |
| T C | 52 | 46.8 | 55 | 51.9 |
| T D | 13 | 11.7 | 16 | 15.1 |
| **Sataloff’s classification N** |  |  |  |  |
| . | 9 | 8.1 | 13 | 12.3 |
| N A | 8 | 7.2 | 9 | 8.5 |
| N B | 40 | 36.0 | 34 | 32.1 |
| N C | 28 | 25.2 | 18 | 17.0 |
| N D | 26 | 23.4 | 32 | 30.2 |

.: not available

**Supplemental table 2**

| **Pathology/Biomarker status** | **RFS points** | **Anastrozole** | **Fulvestrant** |
| --- | --- | --- | --- |
| **pT** |  |  |  |
| . | . | 2 | 13 |
| pT1/T2 | 0 | 100 | 77 |
| pT3/T4 | 3 | 9 | 16 |
| **pN** |  |  |  |
| . | . | 2 | 11 |
| Negative | 0 | 54 | 42 |
| Positive | 3 | 55 | 53 |
| **KI67 level** |  |  |  |
| . | . | 17 | 21 |
| 0%-2.7% | 0 | 34 | 34 |
| >2.7%-7.3% | 1 | 28 | 22 |
| >7.3%-19.7% | 1 | 17 | 18 |
| >19.7%-53.1% | 2 | 15 | 9 |
| >53.1% | 3 | . | 2 |
| **ER-status Allred score** |  |  |  |
| . | . | 8 | 17 |
| 0-2 | 3 | . | 1 |
| 3-8 | 0 | 103 | 88 |
| **PEPI score** |  |  |  |
| . |  | 19 | 28 |
| 0 |  | 17 | 10 |
| 1 |  | 21 | 17 |
| 2 |  | 3 | 8 |
| 3 |  | 15 | 13 |
| 4 |  | 19 | 14 |
| 5 |  | 9 | 1 |
| 6 |  | 2 | 7 |
| 7 |  | 3 | 8 |
| 8 |  | 3 | . |
| **PEPI group** |  |  |  |
| Not calculated |  | 19 | 28 |
| I |  | 17 | 10 |
| II |  | 39 | 38 |
| III (≥4) |  | 36 | 30 |
