# Supplementary material for: Predictive factors of 5-year relapse-free survival in HR+/HER2- breast cancer patients treated with neoadjuvant endocrine therapy: pooled analysis of two phase 2 trials
Source: Br J Cancer. 2020 Jan 31;122(6):759–65. doi: 10.1038/s41416-020-0733-x (PMC7078275; doi:10.1038/s41416-020-0733-x)
Supplement: Supplementary file 2 — Supplementaryi legends [file 41416_2020_733_MOESM2_ESM.docx]

**Supplemental Figure 1 : Relapse-Free survival by PEPI group**.

**Supplemental table 1: Distribution of the population according to the Sataloff classification**.

**Supplemental table 2: Breakdown for the calculated PEPI score for Anastrozole and Fulvestrant.**
